# Predictors of loss to follow-up among adult tuberculosis patients in Southern Ethiopia: a retrospective follow-up study

**DOI:** 10.1186/s12889-022-13390-8

**Published:** 2022-05-14

**Authors:** Desta Watumo, Melkamu Merid Mengesha, Tesfaye Gobena, Mathewos Alemu Gebremichael, Degu Jerene

**Affiliations:** 1 Hosana Health Science College, Hosana, Ethiopia; 2 Epidemiology and Biostatistics Unit, School of Public Health, College of Medicine and Health Sciences, Arba Minch University, Arba Minch, Ethiopia; 3 Department of Environmental Health Sciences, College of Health and Medical Sciences, Haramaya University, Harar, Ethiopia; 4 KNCV Tuberculosis Foundation, Hague, The Netherlands

**Keywords:** Loss to follow-up, Distance to health facility, Adults, Tuberculosis, Southern Ethiopia

## Abstract

**Background:**

Loss to follow-up (LTFU) from tuberculosis (TB) treatment and care is a major public health problem as patients can be infectious and also may develop a multi-drug resistant TB (MDR-TB). The study aimed to assess whether LTFU differs by the distance TB patients travelled to receive care from the nearest health facility.

**Methods:**

A total of 402 patient cards of TB patients who received care were reviewed from March 1–30, 2020. The Kaplan-Meir curve with the Log-rank test was used to compare differences in LTFU by the distance travelled to reach to the nearest health facility for TB care. The Cox proportional hazard regression model was used to identify predictors. All statistical tests are declared significant at a *p*-value< 0.05.

**Results:**

A total of 37 patients were LTFU with the incidence rate of 11.26 per 1000 person-months of observations (PMOs) (95% CI: 8.15–15.53). The incidence rate ratio was 12.19 (95% CI: 5.01–35.73) among the groups compared (those who travelled 10 km or more versus those who travelled less than 10 km). Age ≥ 45 years (aHR = 7.71, 95% CI: 1.72, 34.50), educational status (primary schooling, aHR = 3.54, 95% CI: 1.49, 8.40; secondary schooling, aHR = 2.75, 95% CI: 1.08, 7.03), lack of family support (aHR = 2.80, 95% CI: 1.27, 6.19), nutritional support (aHR = 3.40, 95% CI:1.68, 6.89), ≥ 10 km distance to travel to a health facility (aHR = 6.06, 95% CI: 2.33, 15.81) had significantly predicted LTFU from TB treatment and care.

**Conclusions:**

LTFU from adult TB care and treatment was 12 times higher among those who travelled ≥10 km to reach a health facility compared to those who travelled less. To retain adult TB patients in care and ensure appropriate treatment, health professionals and other stakeholders should give due attention to the factors that drive LTFU. We suggest identifying concerns of older patients at admission and those who travel long distance and establish social support platforms that could help people to complete TB treatment.

**Supplementary Information:**

The online version contains supplementary material available at 10.1186/s12889-022-13390-8.

## Background

Tuberculosis (TB) is a chronic infectious disease which is caused by *Mycobacterium Tuberculosis*. It typically affects the lungs (pulmonary TB) but also it can affect other parts of the body as well (extra pulmonary TB) [1]. The disease is spread via droplet infection when people with pulmonary TB expel the bacilli while coughing, sneezing, or talking [1].

The burden of TB remained to be a major global public health problem despite highly efficacious treatments and preventive methods have been available for decades [2]. Globally, in 2019, an estimated 10.0 million people fell ill with TB. There were 1.2 million and 208, 000 TB deaths among HIV-negative (human immunodeficiency virus) people and people living with HIV (PLHIV), respectively [3]. Among the 22 high TB-burden countries, Ethiopia ranked tenth globally and fourth in Africa [4]. TB is the leading cause of morbidity, the third cause of hospital admission, and the second cause of death in Ethiopia [2]. An estimated 30,000 deaths per year and more than 80 TB-associated deaths occur every day in Ethiopia, with loss to follow-up (LTFU) playing a role in the burden of mortality [2].

A TB patient under treatment follow-up called LTFU when s/he gets lost before treatment initiation or when the treatment is interrupted for two or more successive months. LTFU patients with TB are fraught with problems, predominantly due to multidrug-resistant tuberculosis (MDR-TB) [5]. The most significant indicator for a high burden of MDR-TB could be LTFU [6]. According to the findings of a recent meta-analysis, the estimate of MDR-TB among new and previously treated patients was 2 and 15%, respectively [5]. LTFU also increases the risk of drug toxicity, treatment failure due to poor drug adherence in a case where death is no reason for LTFU [7]. It also results in poorer health outcomes for the patient and contributes to resource wastages [8].

The proportion of LTFU among TB patients varied across different studies. Research evidence reported in Myanmar indicated a 2.5% incidence of LTFU [9], 5.5% in Ethiopia [10], and as high as 44.9% in Mozambique [11]. A study by Alene et al. based on a big sample where district-level data were obtained from regions in Ethiopia through the health management information system and reported an LTFU rate of 5.5% [10]. Various pocket studies in Ethiopia also documented LTFU in different parts of Ethiopia: 13.5% in Jimma university teaching hospital [12], 17.29 per 1000 person-months of observations (PMOs) in public health facilities of Sheka zone [13], and 9.1% in Jinka [14]. These rates were substantially higher than the WHO recommended target of less than 5% [15].

Different factors that predicted LTFU were identified, including socio-demographic factors like residence, social vulnerability, and age [15, 16], individual and behavioral factors [17], clinical, laboratory, and treatment predictors [18], family care, and support related factors [19]. Though previous research that focused on tuberculosis treatment outcome identified the rate of LTFU, they did not characterize factors associated with LTFU, and the evidence generated was limited either to referral or teaching hospitals [10, 14]. However, locally applicable context-specific knowledge of the timing when TB patients get lost from treatment and the factors that predict LTFU are useful to bridge the development of time-relevant intervention approaches. Therefore, this study aimed to assess the incidence and predictors of LTFU among adult tuberculosis patients who received treatment at Gibe Woreda public health facilities, Hadiya zone, southern Ethiopia.

## Methods

### Study setting and period

Data for this study were extracted from March 1 to March 30, 2020, from records of TB patients who enrolled in TB care in four public health facilities (three health centers and one primary hospital) between June 20, 2016, and June 07, 2019, in Gibe Woreda, Hadiya zone, southern Ethiopia. Gibe Woreda has a total population of 141,061, among whom 50.5% were female [20]. The public health facilities provide services in various outpatient and inpatient departments and disease prevention and control activities. Diagnosing and treating TB in Ethiopia is based on Ethiopia’s national TB treatment guidelines [21]. This study was conducted in the Primary Health care Units including the health center and primary hospital. When this study was conducted, Gibe Woreda had three health centers and one primary hospital. The facilities were Homecho Primary Hospital, Omochora Health Centre, Megacho Health Centre, and Amboro Health Centre. Homecho Primary Hospital and Omochora Health Centre independently perform TB diagnosis and initiate directly observed treatment, short-course (DOTS). Whereas, the Megacho and Amboro Health Centres receive TB confirmed referral cases from the nearest highest facilities for DOTS.

### Study design and eligible population

A retrospective follow-up study was conducted based on the record reviews of patients enrolled on first-line TB treatment under DOTS between June 20, 2016, and June 07, 2019. In this study, all adult TB patients (≥15 years old) who had a known TB outcome and registered on TB logbooks in public health facilities in Gibe Woreda were considered eligible. Patients whose records did not include treatment outcomes or whose patient cards indicated they have transferred out to another health facility were excluded. The study cohort was then categorized into two groups based on the main exposure variable to LTFU, which was the distance that patients had to travel from their permanent residential address to reach a health facility providing TB care. We selected distance traveled as the main exposure variable based on the evidence that it strongly predicted LTFU from TB care [13].

### Ethical considerations

All methods in the study were performed following the relevant guidelines and regulations, e.g., the Declaration of Helsinki. The ethical approval is obtained from the Haramaya University College of Health and Medical Sciences Institutional Health Research Ethics Review Committee (IHRERC) with a reference number IHRERC/025/2020. As the study was based on secondary data, on behalf of the patients who received TB care in the selected facilities, informed consent was obtained from the health facility heads to access patient records. Data were collected anonymously to ensure confidentiality.

### Sample size determination and sampling techniques

**Fig. 1:**
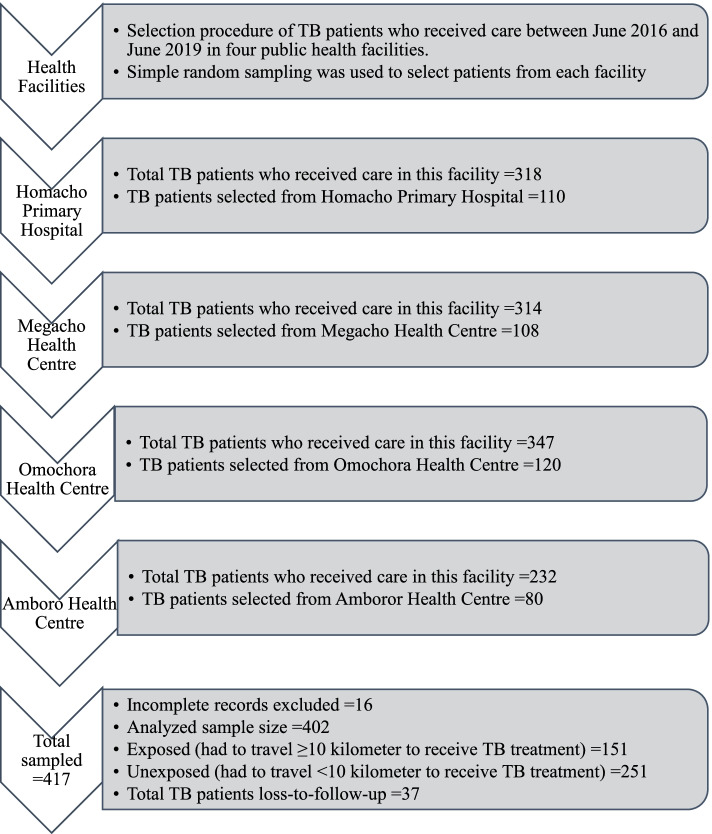
Flow chart depicting how tuberculosis patients’ records from each facility are selected. Based on the number of TB patients enrolled during the study period, June 2016 to June 2019, the sample size was allocated proportionally to the studied health facilities (one primary hospital and three health centers). The distance patients had to travel to a health facility to receive care was the primary exposure variable to the outcome variable, loss to follow-up. TB = Tuberculosis

The sample size was estimated based on a Log-rank test comparing two survival curves using Schoenfed’s method in Stata version 16. Adult TB patients who traveled a distance of over 10 km [13] to reach the nearest health facility to receive TB care constituted the exposed group; those who traveled a distance fewer than 10 km constituted the control groups. The assumptions considered were 5% significance level, 90% power, 1:2 allocation ratio (exposed to the unexposed group), and a reference hazard ratio of 1.4 [13]. Accordingly, the minimum calculated sample size was 397 (149 in the exposed group versus 248 in the unexposed group). Adding 5% for incomplete records separately for each group, the minimum sample size considered was 418 (157 in the exposed versus 261 in the unexposed group). After assigning the minimum calculated sample size proportionally to each of the selected public health facilities based on the size of TB patients enrolled during the study period, a simple random sampling method was used to select individual records of TB patients to be reviewed (Fig. 1).

### Data collection procedure and measurement of study variables

The Data collection tools are developed based on the variables available in the registration logbook and patient card. The data extraction formats were developed based on the variables recorded in these sources. Two diploma nurses in each of the TB clinics collected the data, and the overall data collection was supervised by one of the investigators. The data extraction tool included variables on socio-demographic characteristics (age, sex, residence, marital status, occupational status, educational status, and religion), distance traveled, functional status, type of TB, previous TB treatment, DOTS follow-up center, treatment outcome, ART medication status, family support, and nutrition support.

Distance traveled (the average distance that a patient from the same sub-district traveled) to reach the nearest health facility to receive TB care irrespective of the means of transport was coded as 1″ < 10 km” and 2″ ≥ 10 km”. The measured values of this variable are recorded in the registration logbook. As health professionals working in the TB caregiving facility frequently went out for supportive supervision to their catchment sub-districts (Kebeles), they have valid information on how far a certain village is located from a given health facility.

Diagnosis and treatment of TB were made following to the Ethiopian National Guidelines on TB, Drug-resistant TB, and Leprosy [22]. The guideline suggests using sputum microscopy as a primary diagnostic tool in the absence of Xpert. Accordingly, a TB patient is said to have a smear-positive pulmonary TB (PTB^+^) if s/he has positive acid-fast bacilli (AFB) results for at least one or two initial sputum specimens by direct microscopy. Diagnosis of smear-negative pulmonary TB (PTB^−^) was established when a patient having symptoms suggestive of TB had two AFB negative test results by direct microscopy, and no response to a course of broad-spectrum antibiotics, and radiological abnormalities consistent with pulmonary TB, and decision by a clinician to treat with a full course of anti-TB or patient whose diagnosis is based on culture positive for *Mycobacterium tuberculosis*. When a patient had culture-proven or histopathologic evidence from a biopsy or strong clinical evidence that TB has affected body organs other than the lungs, s/he is diagnosed with extra-pulmonary tuberculosis (EPTB) and a physician decides to treat the patient with a full course of anti-TB therapy. Patients receive daily rifampicin, pyrazinamide, isoniazid, and ethambutol for 2 months (intensive phase) followed by daily rifampicin and isoniazid for 4 months or more (continuation phase). Five mutually exclusive treatment outcomes are documented including cured (confirmed smear-negative in the last month of treatment and on at least one previous occasion), treatment completed (a patient completed treatment and had no evidence of failure but without records to evidence cure), treatment failure (a patient whose sputum smear or culture is positive at month 5 or later during treatment), died (patient who dies during TB treatment), and LTFU (a patient who has been on TB treatment for at least 4 weeks and whose treatment was interrupted for eight or more consecutive weeks). Treatment success was finally defined as a sum of cured and completed treatments [22].

Nutrition support was guided by the cut-off points for body mass index (BMI) indicated in the National Guidelines [22]. Nutritional support is recommended for adults identified at admission with severe acute malnutrition (SAM) (BMI < 16 Kg/m2) and moderate acute malnutrition (MAM) (16 Kg/m^2^ ≥ BMI < 17 Kg/m^2^) and had TB/HIV co-infections. Plumpy nut (an energy-dense fortified therapeutic food designed for the treatment of SAM) or plumpy sup (an energy-dense fortified therapeutic food designed for the treatment of MAM) based nutrition intervention was provided for 6 months (Plumpy nut for the first 3 months and Plumpy sup for another 3 months) for TB patients with SAM and Plumpy sup for 3 months for TB patients who had MAM at baseline [22].

### Data management and statistical analysis

Data were checked for completeness and consistency and entered into Epi Data version 3.1 and exported to Stata version 16.0 for data management and statistical analysis. Descriptive statistics were computed to summarize findings and reported using numerical summary measures for continuous variables and percentages for categorical variables. Data for dates are collected and documented in the Ethiopian calendar. These have been changed to the Gregorian calendar by adding 2806 days to the dates documented in the Ethiopian calendar. The origin of time was the date of admission and the endpoint was the date event occurred or the date censoring occurred. After declaring our data in Stata as survival data, we obtained person-time months of observation, incidence rates, incidence rate ratio, compared these values by the grouping variable, distance traveled to health facilities. The event of interest was LTFU and other censored outcomes include cure, treatment completion, treatment failure, and death. The Kaplan-Meier survival curves together with the log-rank test were presented to display whether there was a significant difference in survival probability among adult TB patients by the distance they traveled to a health facility to receive TB care. The cumulative survival probabilities are presented in Life Table. To identify variables that best predicted LTFU, the Cox regression model was used. Before running the bivariable model, we run the null model to see how each variable improved the model compared to the null model. A variable with *P* < 0.25 and added improvement to the model prediction was entered into a multivariable Cox proportional hazards regression model. The Cox proportional hazard regression assumptions were checked using the Schoenfeld residual test which tests the correlation between residuals and the survival time; *P*-value greater than 0.05 indicates that the proportional hazard regression assumption was met. Using the post-estimation ‘phtest’ command in Stata, we obtained *P*-values well above the significance test both for the global test (chi2 = 11.98, *P*-value = 0.447) and also for each of the covariates (the minimum and maximum *P*-values obtained were 0.078 versus 0.948). All statistical tests that compared survival curves and predicted LTFU were declared significant at *P*-value< 0.05.

## Results

### Socio-demographic characteristics of the study participants

A total of 402 patients who initiated TB treatment between June 2016 and June 2019 were included in this analysis. Males accounted for 55.5% of study participants, and 53% were aged 35 years and above with a mean (standard deviation) age of 39 years (±16.8). The majority of participants, 62%, were married, and also 62% lived within a 10-km radius from the health facilities in Gibe Woreda (Table 1).

**Table 1:** Socio-demographic characteristics of adult TB infected patients by distance from TB clinics in Gibe Woreda, Hadiya zone southern Ethiopia from June 20, 2016, and June 07, 2019

Characteristic	Categories	Total (*N* = 402), (%)	Exposed^a^ (*N* = 151), n (%)	Not exposed^b^ (*N* = 251), n (%)
Age (Years)	15–24	103 (25.6)	25(16.6)	78(31.1)
	25–34	81 (20.2)	28(18.5)	53 (21.1)
	35–44	74 (18.4)	27(17.9)	47(18.7)
	≥45	144 (35.8)	71 (47.0)	73 (29.1)
Sex	Male	223 (55.5)	75 (49.7)	148 (59.0)
	Female	179 (44.5)	76 (50.3)	103 (41.0)
Marital status	Married	248 (61.7)	94(62.3)	154(61.4)
	Single	146 (36.3)	53 (35.1)	93 (37.1)
	Other^c^	8 (2.0)	4 (2.6)	4 (1.6)
Educational status	None	137 (34.1)	59(39.1)	78(31.1)
	Primary	110 (27.4)	34(22.5)	76(30.3)
	Secondary	103 (25.6)	39 (25.8)	64(25.5)
	Tertiary	52 (12.9)	19 (12.6)	33 (13.2)
Occupational status	Civil servant	35 (8.7)	13(8.6)	22(8.8)
	Farmer	137 (34.1)	47(31.1)	90(35.9)
	Merchant	73 (18.2)	30 (19.9)	43(17.1)
	Housewife	47 (11.7)	22 (14.6)	25 (10.0)
	Student	102 (25.4)	34 (22.5)	68 (27.1)
	Daily labourer	8 (2.0)	5 (3.3)	3 (1.2)
Residence	Rural	334 (83.1)	104(66.9)	230(91.6)
	Urban	68 (16.9)	47 (33.1)	21 (8.4)
Religion	Protestant	287 (71.4)	112(74.2)	175(69.7)
	Orthodox	106 (26.4)	35 (23.2)	71(28.3)
	Muslim	9 (2.2)	4 (2.6)	5 (2.0)

^a^ ≥ 10 km

^b^ < 10 km

^c^separated, divorced, and widowed

### Clinical characteristics and treatment outcome

The majority of the patients, 69.9%, had their DOTS follow-up at the health centers. On admission to the TB-clinics, 19.9% of patients were screened to receive nutritional support. Concerning baseline functional status, 61.9% of study participants were able to perform their routine activities. On admission, 46.8% of patients were diagnosed with new pulmonary negative, 33.1% new pulmonary positive, and 20.2% had extrapulmonary TB. Eighty-six percent of TB patients were screened for HIV, and 2.7% were on ART. Almost all TB patients, 97.5%, had no history of previous TB treatment and 87% reported that they received family support during their treatment. 9.2% of adult TB patients were LTFU (Table 2).

**Table 2:** Clinical, laboratory, and treatment-related characteristics of adult TB patients by distance from TB clinics in Gibe Woreda public health facilities, Hadiya zone, southern Ethiopia

Characteristics	Categories	Total (*N* = 402), n (%)	Exposed^a^ (*N* = 151), n (%)	Not exposed^b^ (*N* = 251), n (%)
Baseline functional status	Working	249 (61.9)	100(66.2)	149(59.4)
	Ambulatory	144 (35.8)	45(29.8)	99(39.4)
	Bedridden	9 (2.2)	6 (4.0)	3(1.2)
Types of TB on admission	New pulmonary TB	133 (33.1)	52 (34.4)	81 (32.3)
	New pulmonary negative	188 (46.8)	70 (46.4)	118 (47.0)
	Extra pulmonary TB	81 (20.2)	29 (19.2)	52 (20.7)
ART medication	Yes	11 (2.7)	4(2.7)	7(2.8)
	No	391 (97.3)	147 (97.3)	244(97.2)
Previous TB treatment	Yes	10 (2.5)	4(2.7)	6(2.4)
	No	392 (97.5)	147(97.3)	245(97.6)
Family support	Yes	349 (86.8)	111(73.5)	238 (94.8)
	No	53 (13.2)	40 (26.5)	13(5.2)
Nutritional support	Yes	80 (19.9)	35(23)	45(18)
	No	322 (80.1)	116 (77)	206(82)
DOTS follow-up	Hospital	87 (21.6)	31(20.5)	56(22.3)
	Health centre	281 (69.9)	106 (70.2)	175(69.7)
	Health post	34 (8.5)	14 (9.3)	20(8.0)
Treatment outcome	Lost	37 (9.2)	31 (20.5)	6(2.4)
	Censored^c^	365 (90.8)	120(79.5)	245 (97.6)

*ART* antiretroviral therapy, *TB* tuberculosis

^a^ ≥ 10 km

^b^ < 10 km

^c^cured, completed treatment, died, failure, and transferred out

### Incidence Rate of LTFU

All the study participants contributed a total of 3287.37 person-months of observations (PMOs). Of these, 251 the unexposed group of patients, contributed 2308.57 PMOs, whereas 151 patients, the exposed group of patients, contributed 978.8 PMOs. Thirty-seven patients were LTFU, 31 of these were in the exposed group. The overall incidence rate was 11.26 per 1000 PMOs (95% CI: 8.15–15.53) and the incidence rate ratio by the groups compared was 12.19 (95% CI; 5.01–35.73). In terms of the cumulative incidence, the proportion of LTFU among adults who traveled 10 km or more was 20.5% (95% CI: 14.7, 27.8) compared to only 2.4% (95% CI: 1.1, 5.2) among those who traveled fewer than 10 km. The overall cumulative incidence of LTFU among the cohort was 9.2% (95% CI: 6.7, 12.4).

The probability of remaining on treatment after completing the intensive phase of treatment was 0.99 for the unexposed group compared to the exposed group, 0.94. The probability of survival at 5 months of treatment for the exposed cohort was 0.87 compared to 0.98 for the unexposed cohort. Furthermore, only 80% of patients in the exposed cohort completed the usual six-month duration of treatment (Additional file 1). Among the overall LTFU events, 23.7% (95% CI: 12.5, 40.2) occurred during the intensive treatment phase and 76.3% (95% CI: 59.8, 87.5) occurred during the continuation phase.

**Fig. 2:**
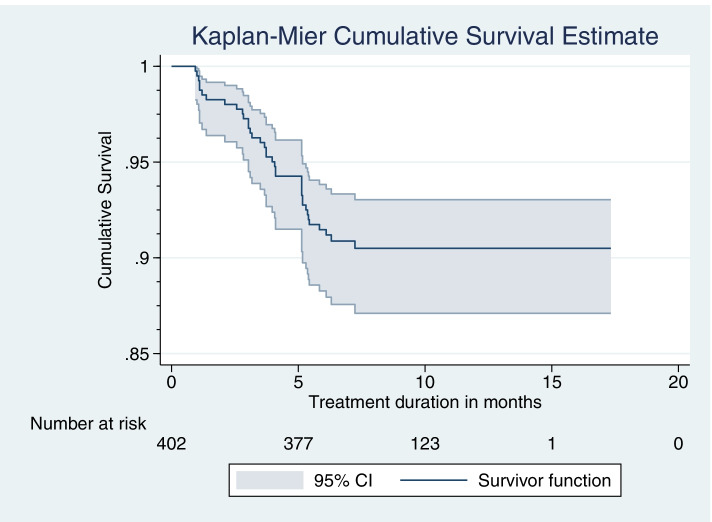
Kaplan-Meier survival estimate of LTFU among adult TB patients on treatment in Gibe Woreda public health facility, Hadiya zone, Southern Ethiopia, from 2016 to 2019

As can be seen from the cumulative survival curve, LTFU was observed until the 7 months of treatment and levels off thereafter where over 90% of participants did not experience the event (Fig. 2).

**Fig. 3:**
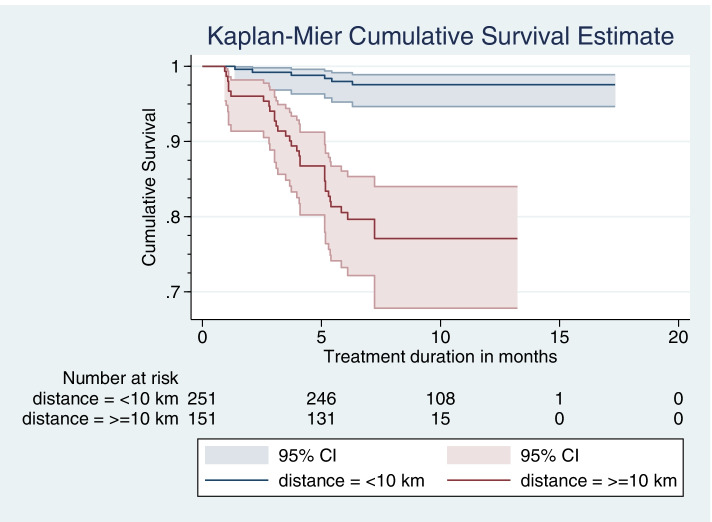
Survival experience of LTFU among adult TB patients on treatment by exposure status, in Gibe Woreda public health facility, Hadiya zone, Southern Ethiopia, from 2016 to 2019

When stratified by distance from a health facility, the proportion experiencing LTFU significantly varies as indicated by the log-rank test (Fig. 3).

### Predictors of LTFU among TB patients

Adjusting for the effect of other variables in the model, adults who traveled ≥10 Kilometers to receive TB care had a 6-times higher risk of LTFU (≥ 10 Kilometers, aHR = 6.06, 95% CI: 2.33, 15.81). Older age was also associated with a 7-fold increase in the risk of LTFU (Age > = 45 years, aHR = 7.71, 95% CI: 1.72, 34.50). Adults who reported that they had no family support in their TB treatment and care had 2.8-times higher risk of LTFU (no family support, aHR = 2.80, 95% CI: 1.27, 6.19). Likewise, compared to adults who had no formal education, those who attended primary or secondary school grades had 2.7 to 3.3 times increased risk of LTFU (primary (1–8 grades), aHR = 3.54, 95% CI: 1.49, 8.41; secondary (9–10 grades), aHR = 2.75, 95% CI: 1.08, 7.03). On contrary, those TB patients who got nutritional support had a 3.3 times higher risk of LTFU (Received nutritional support, aHR = 3.40, 95% CI: 1.68, 6.89) (Table 3).

**Table 3:** Predictors of LTFU among adult TB patients who received TB care in Gibe woreda public health facilities, Hadiya zone, southern Ethiopia (*n* = 402)

Characteristics	LTFU (n, %)	Censored^a^ (n, %)	cHR (95% CI)	aHR (95% CI)
Age in years (Ref.: 15–24 years)	2 (5.4)	101 (27.7)		
25–34 years	2 (5.4)	79 (21.6)	1.28(0.18, 9.09)	1.26 (0.17, 9.07)
35–44 years	2 (5.4)	72 (19.7)	1.41(0.20, 10.0)	1.48 (0.20, 11.00)
> =45 years	31 (83.8)	113 (31.0)	12.38(2.96, 51.75)**	7.71 (1.72, 34.50)*
Gender (Ref.: female)	8 (21.6)	171 (46.8)		
Male	29 (78.4)	194 (53.2)	3.02(1.38,6.61)**	2.32(0.97, 5.55)
Education (Ref.: no formal education)	11 (29.7)	126 (34.5)		
Primary (1–8 grades)	11 (29.7)	99 (27.1)	1.22(0.53, 2.81)	3.54(1.49, 8.41)**
Secondary (9–10 grades)	10 (27.0)	93 (25.5)	1.21 (0.52, 2.86)	2.75(1.08, 7.03)*
Preparatory/College or above	5 (13.5)	47 (12.9)	1.17 (0.41, 3.37)	2.59(0.88, 7.64)
Tuberculosis type on admission (Ref.: pulmonary positive)	18 (48.7)	115 (31.5)		
Pulmonary negative	16 (43.2)	172 (47.1)	0.62(0.32, 1.21)	0.90(0.45, 1.80)
Extra pulmonary	3 (8.1)	78 (21.4)	0.26(0.08, 0.88)*	0.38(0.11, 1.33)
Nutritional support (Ref.: no)	21 (56.8)	301 (82.5)		
Yes	16 (43.2)	64 (17.5)	3.24(1.69, 6.21)***	3.40(1.68, 6.89)**
Family support on TB care (Ref.: yes)	18 (48.7)	331 (90.7)		
No	19 (51.3)	34 (9.3)	8.58 (4.97, 16.36)***	2.80(1.27, 6.19)*
Distance from a health facility (Ref.: < 10 km)	6 (16.2)	245 (67.1)		
≥ 10 km	31 (83.8)	120 (32.9)	10.29(4.27, 24.83)***	6.06(2.33, 15.81)**

*LTFU* Lost to follow-up, *cHR* crude odds ratio, *aHR* adjusted hazard ratio, *CI* confidence interval, *TB* tuberculosis, *Ref.* reference category

^a^cured, treatment completed, died, treatment failure, transferred out

^⁎^*p* value < 0.05

^⁎⁎^*p* value < 0.01

^⁎⁎^*p* value < 0.001

## Discussion

In this study, one in 10 patients became LTFU from TB treatment and care, and long-distance a patient had to travel to a TB clinic was a significant predictor of LTFU. The other factors that predicted LTFU include adults over the age of 45 years, a lower level of education, and a lack of family support. The higher LTFU among patients who received nutritional support in the study setting was an unexpected finding. Our results provide important inputs to tailor interventions to the specific local needs to achieve the goal of ending TB by 2030, emphasizing factors that impact the rate of LTFU of patients on TB treatment [23].

The overall incidence rate of LTFU was 11.26 (95% CI: 8.15–15.53) per 1000 PMOs, and the overall proportion of LTFU was 9.2% (95% CI: 6.7, 12.4). The incidence rate of LTFU in this study was lower compared to the LTFU rate of 27.3 per 1000 PMOs reported in the Sheka zone, South-west Ethiopia [13]. Higher cumulative incidences of LTFU are reported in Jimma University teaching hospital, 13.5% [12], and as high as 20% in Hossana hospital, south Ethiopia [24]. The estimated cumulative incidence of LTFU in this study was consistent with an 8% LTFU after treatment initiation in Pakistan [25] and in Jinka, Southern Ethiopia, where a 9.1% LTFU was reported [14]. However, the LTFU cumulative incidence of 9.2% in this study was higher compared to the national LTFU level of 5.5% in Ethiopia [10]. The observed differences could be due to differences in the time points the studies were conducted and could be due to changes prompted by the 2016 END TB strategy. For example, high reports of LTFU in Akessa et al. [12] and Wondale et al. [14] were reported among studies conducted before the implementation of the END-TB strategy. Other possible explanations could be the difference in case-finding and surveillance, assistance given to patients, and other environmental factors.

In our study, 76% (95% CI: 59.8, 87.5) of LTFU cases occurred during the continuation phase, and the rest, 24% (95% CI: 12.5, 40.2), happened during the intensive phase of treatment. This finding was in agreement with findings from a study in Jinka, Southern Ethiopia, where 81% of the TB patients become LTFU in the continuation phase [14]. This is could be due to that patients may feel that they were cured after the intensive phase of treatment or it may be because of the reason that sputum follow-up was not performed or documented during follow-up. It is important hence that patients should receive a continued reinforcement that emphasizes treatment completion despite that they feel better after the intensive period of treatment.

This study revealed that there was a significant association between distance travelled to reach the nearest health facility and the rate of LTFU of TB patients. Patients who travelled a long-distance (≥ 10 km) had a five times higher risk of LTFU compared to those who travelled less than 10 km. This finding was consistent with the finding reported by Shewano et al. from Sheka Zone in southwestern Ethipia [13]. A large study by Kibuule et al. in Namibia also reported that proximity to DOTS access points was related to a lower level of LTFU [26]. In addition to the distance travelled to reach the nearest health facility to access care, a study in central India also mentioned the contribution of seasons of the year to LTFU [27]. Furthermore, economic constraints of transportation costs may also have justified how distance was associated with increasing LTFU [28]. This finding highlights the need to prioritize structural barriers to and social determinants of TB care that are often overlooked in conventional TB care. Patient LTFU that still occurs at distant locations despite a health extension worker(s) pay a house-to-house visit in each rural village calls for alternative solutions to bring the services closer to the community.

Compared to younger adults aged 15–24 years old, patients who were 45 years or older had seven times higher risk of LTFU from TB care. Several studies have previously reported consistent findings. Sheweno et al., for example, reported that for a unit increase in age, the risk of LTFU from TB care increased by 70% [13]. Similar findings were also reported by Lwin et al. in Myanmar [9], Wen et al. in China [29], and Ejeta et al. in the Gambella region of Ethiopia [30]. A possible explanation could be that with an advance in age, as this study was conducted in a rural community, adults would get weaker to travel long distances on foot (or unable to pay to take transport if any) or may have other comorbidities preventing them from catching their follow-up visits to TB clinic.

Education was also identified as one of the individual factors that increased the risk of LTFU. Adult patients who completed primary school (grades 1–8 in the Ethiopian context) or those who did not go beyond grade 10 had a strong positive association with LTFU. Similar studies reported that a few years of schooling and poor knowledge of TB were associated with an increased risk of LTFU [31–33]. While Mukhtar et al. [32] and Viana et al. [33] reported the association of lower educational status and a higher risk of LTFU, Belchior et al. [31] described that scarce knowledge on TB is associated with LTFU. It’s also likely that this sub-group of patients may hold misconceptions or easily get deceived by misinformation about TB. Therefore, providing health education to patients that match their level of understanding could be helpful.

Interestingly we found that adult TB patients who received nutrition support had an increased risk of LTFU. It was against our expectation that nutritional support could improve weight gain, adherence, and lead to a fast recovery. This expectation was also evidenced in previous reports [34–37] and that baseline body weight was a significant predictor of LTFU [38, 39]. Other reports, however, pointed out that despite nutritional support contributing to improving patient nutritional status on the course of treatment, there was no sufficient evidence whether or not nutritional support is associated with a better TB treatment outcome [35]. The possible explanation for our finding might be that these patients had a more advanced disease condition requiring nutritional support, as a result, of which they may have died in the community. Similarly, it was also possible that the nutritional support has led to a rapid recovery resulting in a misperception that the incident episode of TB had resolved and failed to continue with their clinic visit for treatment. Furthermore, they might have been unable to come to health facilities due to high transport costs. This finding suggests that mere nutritional support may not be sufficient to improve treatment outcomes without addressing the root causes that led to nutritional deprivation in the first place. A study in Kenya, for example, reported incorporating nutritional counselling for TB patients receiving food support was associated with a further reduction in LTFU [37].

Lack of family support to TB patients was associated with over a two-fold increase in the risk of LTFU compared with those who had family support during treatment. Different studies have emphasized the importance of family support and also psychological support from healthcare workers to reduce or prevent LTFU from TB care and treatment [28, 40, 41]. In countries like Ethiopia**,** where the age-dependency ratio is high [42], supports including finance from active family members is essential for successful treatment completion. Family support should not be undervalued as patients need the support of someone closer to them on transport cost, collection of medication, supervision on the taking of medication, reminding of appointments, and emotional support.

Our study was not without limitation that the data analyzed was restricted to variables available on patient records where the opportunity to control potential confounders was less likely. The exclusion of incomplete records might have affected the estimated rate of LTFU. Regarding the effect of differing follow-up periods on the incidence of LTFU, we acknowledge that prolonged follow-up time beyond the recommended six-month to some of the study subjects may have over-estimated the incidence of LTFU. Furthermore, as nurses working in health facilities selected for the study collected the data, bias could be introduced as they may fail to objectively extract the negative outcomes of patients treated in their facility. Finally, LTFU occurring due to dissatisfaction with the healthcare system cannot be ruled out. Thus, this finding should be interpreted with these limitations in mind.

## Conclusion

This study reported a high incidence of LTFU and prevalence higher than the national average in Ethiopia. Factors significantly associated with an increase in the risk of LTFU include patients who had to travel a long-distance to TB clinic; adults aged 45 years or older, low educational status, lack of family support, and availability of nutritional support. For TB treatment completion, we recommend that health professionals and other stakeholders working to stop TB should closely monitor to understand the problems of older patients and those who travel long-distance. Furthermore, it is important to establish social support and awareness creation platforms to help reduce misunderstandings that may contribute to dropouts. Finally, further investigations are needed to complete the understanding of why TB patients get lost from treatment and also investigate how nutritional support affects TB treatment completion.

## Supplementary Information

**Additional file 1.** Life table analysis of LTFU among TB infected adult patients on TB care in Gibe Woreda public health facility Hadiya zone, Southern Ethiopia from June 2016 to June 2019.

## Data Availability

All data pertaining to the findings are presented in this paper. However, the data can be obtained from the corresponding author any time on reasonable request.

## Abbreviations

AFB: Acid-Fast Bacilli
aHR: Adjusted Hazard Ratio
ART: Antiretroviral Therapy
BMI: Body Mass Index
cHR: Crude Hazard Ratio
CI: Confidence Interval
DOTS: Directly Observed Treatment, Short Course
EPTB: Extra Pulmonary Tuberculosis
HIV: Human Immunodeficiency Virus
LTFU: Lost to Follow-Up
MAM: Moderate Acute Malnutrition
MDR-TB: Multi-Drug Resistant Tuberculosis
PMOs: Person Months of Observations
PTB: Pulmonary Tuberculosis
SAM: Sever Acute Malnutrition
TB: Tuberculosis
