# Supplementary material for: Predictors of loss to follow-up among adult tuberculosis patients in Southern Ethiopia: a retrospective follow-up study
Source: BMC Public Health. 2022 May 14;22:976. doi: 10.1186/s12889-022-13390-8 (PMC9107690; doi:10.1186/s12889-022-13390-8)
Supplement: Supplementary file 1 — Additional file 1. Life table analysis of LTFU among TB infected adult patients on TB care in Gibe Woreda public health facility Hadiya zone, Southern Ethiopia from June 2016 to June 2019. [file 12889_2022_13390_MOESM1_ESM.docx]

Additional file 1. Life table analysis of LTFU among TB infected adult patients on TB care in Gibe Woreda public health facility Hadiya zone, Southern Ethiopia from June 2016 to June 2019

| Interval | Beginning total | Event | Censored | Survival probability | SE^†^ | 95% CI |
| --- | --- | --- | --- | --- | --- | --- |
| Unexposed group | | | | | | |
| 1-2 | 251 | 1 | 0 | 0.9960 | 0.0040 | 0.9721, 0.9994 |
| 2-3 | 250 | 1 | 0 | 0.9920 | 0.0056 | 0.9685, 0.9980 |
| 3-4 | 249 | 1 | 2 | 0.9880 | 0.0069 | 0.9634, 0.9961 |
| 5-6 | 246 | 2 | 6 | 0.9799 | 0.0089 | 0.9524, 0.9916 |
| 6-7 | 238 | 1 | 18 | 0.9756 | 0.0098 | 0.9465, 0.9890 |
| 7-8 | 219 | 0 | 30 | 0.9756 | 0.0098 | 0.9465, 0.9890 |
| 8-9 | 189 | 0 | 16 | 0.9756 | 0.0098 | 0.9465, 0.9890 |
| 9-10 | 173 | 0 | 65 | 0.9756 | 0.0098 | 0.9465, 0.9890 |
| 10-11 | 108 | 0 | 61 | 0.9756 | 0.0098 | 0.9465, 0.9890 |
| 11-12 | 47 | 0 | 37 | 0.9756 | 0.0098 | 0.9465, 0.9890 |
| 12-13 | 10 | 0 | 8 | 0.9756 | 0.0098 | 0.9465, 0.9890 |
| 13-14 | 2 | 0 | 1 | 0.9756 | 0.0098 | 0.9465, 0.9890 |
| 17-18 | 1 | 0 | 1 | 0.9756 | 0.0098 | 0.9465, 0.9890 |
| Exposed group | | | | | | |
| 0-1 | 151 | 1 | 0 | 0.9934 | 0.0066 | 0.9539, 0.991 |
| 1-2 | 150 | 5 | 0 | 0.9603 | 0.0159 | 0.9137, 0.9820 |

Additional file, Table 1. Cont’d.

| Interval | Beginning total | Event | Censored | Survival probability | SE | 95% CI |
| --- | --- | --- | --- | --- | --- | --- |
| 2-3 | 145 | 3 | 0 | 0.9404 | 0.0193 | 0.8886, 0.9685 |
| 3-4 | 142 | 8 | 0 | 0.8874 | 0.0257 | 0.8252, 0.9285 |
| 4-5 | 134 | 3 | 0 | 0.8874 | 0.0257 | 0.8252, 0.9285 |
| 5-6 | 131 | 9 | 23 | 0.8675 | 0.0276 | 0.8023, 0.9124 |
| 6-7 | 99 | 1 | 51 | 0.8022 | 0.0330 | 0.7278, 0.8583 |
| 7-8 | 47 | 1 | 23 | 0.7913 | 0.0343 | 0.7142, 0.8498 |
| 8-9 | 23 | 0 | 7 | 0.7690 | 0.0399 | 0.6791, 0.8367 |
| 9-10 | 16 | 0 | 1 | 0.7690 | 0.0399 | 0.6791, 0.8367 |
| 10-11 | 15 | 0 | 7 | 0.7690 | 0.0399 | 0.6791, 0.8367 |
| 11-12 | 8 | 0 | 6 | 0.7690 | 0.0399 | 0.6791, 0.8367 |
| 12-13 | 2 | 0 | 1 | 0.7690 | 0.0399 | 0.6791, 0.8367 |
| 13-14 | 1 | 0 | 1 | 0.7690 | 0.0399 | 0.6791, 0.8367 |

^†^SE=Standard error; LTFU=loss to follow-up; TB=Tuberculosis; Exposure status was based distance travelled to the nearest health facility to receive TB care: exposed≥10 kilometers and unexposed<10 kilometers.
